# SWCNT Photocatalyst for Hydrogen Production from Water upon Photoexcitation of (8, 3) SWCNT at 680-nm Light

**DOI:** 10.1038/srep43445

**Published:** 2017-03-06

**Authors:** Noritake Murakami, Yuto Tango, Hideaki Miyake, Tomoyuki Tajima, Yuta Nishina, Wataru Kurashige, Yuichi Negishi, Yutaka Takaguchi

**Affiliations:** 1 Graduate School of Environmental and Life Science; Okayama University; 3-1-1 Tsushima-Naka, Kita-ku, Okayama 700-8530, Japan; 2 Graduate School of Sciences and Technology for Innovation; Yamaguchi University, 2-16-1 Tokiwadai, Ube, Yamaguchi 755-8611, Japan; 3 Research Core for Interdisciplinary Sciences; Okayama University, 3-1-1 Tsushima-Naka, Kita-ku, Okayama 700-8530, Japan; 4 Department of Applied Chemistry; Faculty of Science Division I; Tokyo University of Science, 1-3 Kagurazaka, Shinjuku-ku, Tokyo 162-8601, Japan

## Abstract

Single-walled carbon nanotubes (SWCNTs) are potentially strong optical absorbers with tunable absorption bands depending on their chiral indices (*n, m*). Their application for solar energy conversion is difficult because of the large binding energy (>100 meV) of electron-hole pairs, known as excitons, produced by optical absorption. Recent development of photovoltaic devices based on SWCNTs as light-absorbing components have shown that the creation of heterojunctions by pairing chirality-controlled SWCNTs with C_60_ is the key for high power conversion efficiency. In contrast to thin film devices, photocatalytic reactions in a dispersion/solution system triggered by the photoexcitation of SWCNTs have never been reported due to the difficulty of the construction of a well-ordered surface on SWCNTs. Here, we show a clear-cut example of a SWCNT photocatalyst producing H_2_ from water. Self-organization of a fullerodendron on the SWCNT core affords water-dispersible coaxial nanowires possessing SWCNT/C_60_ heterojunctions, of which a dendron shell can act as support of a co-catalyst for H_2_ evolution. Because the band offset between the LUMO levels of (8, 3)SWCNT and C_60_ satisfactorily exceeds the exciton binding energy to allow efficient exciton dissociation, the (8, 3)SWCNT/fullerodendron coaxial photocatalyst shows H_2_-evolving activity (QY = 0.015) upon 680-nm illumination, which is E_22_ absorption of (8, 3) SWCNT.

Visible light-induced water splitting has received considerable attention in terms of solar energy conversion and hydrogen energy storage^1^,^2^,^3^,^4^,^5^,^6^. Although various semiconducting materials for H_2_-evolution photocatalysts have been reported^7^,^8^,^9^, examples of efficient H_2_ production using illumination wavelengths over 600 nm are very limited. For example, Domen and co-workers reported that g-C_3_N_4_ photosensitized by magnesium(II) phthalocyanine showed photocatalytic H_2_ production activity under visible light irradiation up to 700 nm, but with a rather low quantum efficiency (0.07% at 660 nm)^10^. Recently, Li and co-workers improved this phthalocyanine/g-C_3_N_4_ system by employing a highly asymmetric zinc(II) phthalocyanine derivative to achieve efficient photocatalytic H_2_ production with an apparent quantum yield (AQY) of 1.85% at 700 nm^11^. Because the main component of the solar spectrum consists of visible light (400 < λ < 800 nm), the unexplored region of visible light, from 600 nm to 800 nm, should be fully exploited by employing a new class of photocatalysts. Previously, we described the fabrication and efficient photoinduced electron transfer processes of single-walled carbon nanotube (SWCNT)/anthryl dendron^12^,^13^ and SWCNT/fullerodendron^12^,^14^ supramolecular nanocomposites, of which coaxial nanowire structures provide a photofunctional interface between the SWCNT core and the dendron shell. Since recent studies indicated that coaxial nanowire structures could improve carrier collection and overall efficiency with respect to bulk semiconductors of the same materials^15^, we have explored photosensitized hydrogen evolution from water under visible light irradiation (450 nm) using our coaxial supramolecular photosensitizers possessing a SWCNT core^16^, such as SWCNT/fullerodendron or SWCNT/fullerodendron/SiO_2_, in the presence of methyl viologen dication (an electron relay) and platinum nanoparticles (a co-catalyst). Interestingly, these three-component systems, i.e., a H_2_-evolution photocatalytic system consisting of a photosensitizer, an electron relay, and a co-catalyst, showed very high quantum yields of 0.28 for SWCNT/fullerodendron and 0.31 for SWCNT/fullerodendron/SiO_2_, respectively, under 450 nm light illumination. In addition, we succeeded in the construction of a co-catalyst interconnecting system, a SWCNT/fullerodendron/Pt(II) complex showing efficient H_2_ evolution (1.2 μmol/h) under visible light irradiation (>422 nm) in the absence of an electron relay molecule^17^. However, the absorption bands of SWCNTs cannot be used effectively because HiPco tubes, of which the semiconducting component absorbs light at wavelengths over 800 nm, were used as the core of these coaxial nanophotosensitizers^18^. From the viewpoint of better performance of a H_2_ evolution photocatalyst that is responsive to illumination wavelengths from 600 nm to 800 nm, SWCNT cores having an appropriate band structure are required for the construction of a coaxial nanophotosensitizer. Since the fact that the electronic properties of SWCNTs depend on the chiral indices (*n, m*) has clearly been demonstrated by Nakashima^19^, several attempts have been made to explore the effect of the chirality of SWCNTs on the efficiency of photoinduced electron transfer with C_60_. For example, Isborn *et al*. demonstrated that the likelihood of exciton separation and charge transfer from SWCNTs to C_60_ increased in the order of three SWCNT chiralities, (9, 7) < (7, 6) < (6, 5) by the use of SWCNT/C_60_ photovoltaics and DFT band structure calculations^20^. Flavel *et al*. reported that the diameter limit of SWCNTs in SWCNT/C_60_ solar cells was (8, 6)SWCNT with a diameter of 0.95 nm^21^. Recently, Blackburn *et al*. described an optimum LUMO offset between SWCNTs and C_60_ of approximately 130 meV, which is satisfied for small diameter nanotubes, such as (8, 3), (9, 1), or (6, 5)^22^. These circumstances prompt us to investigate the fabrication of photosensitizer/co-catalyst interconnecting systems having coaxial nanowire structures by employing small diameter SWCNTs. Fortunately, recent developments in the large-scale single-chirality separation of SWCNTs^23^,^24^,^25^ have provided us with the opportunity to use (6, 5)-enriched SWCNTs, which contain only small diameter SWCNTs, such as (6, 5), (7, 5), (7, 6) and (8, 3). Here, we describe the first example of photosensitized hydrogen evolution from water triggered by the photoexcitation of SWCNTs. In particular, highly efficient H_2_ evolution was observed by selective photoexcitation of (8, 3) SWCNT upon 680-nm illumination.

## Results and Discussion

**Figure 1:**
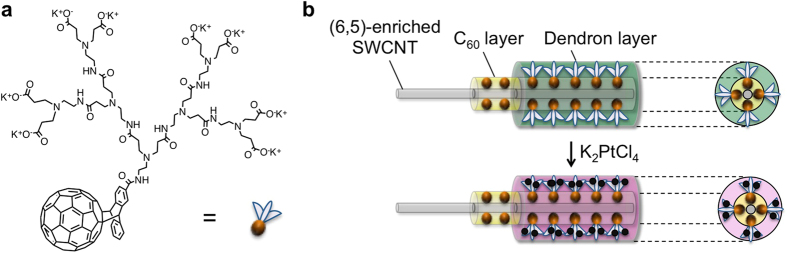
Fabrication of (6, 5)-enriched SWCNT/fullerodendron/Pt(II) coaxial photocatalyst. (**a**) Molecular structure of fullerodendron. (**b**) Schematic illustration of (6, 5)-enriched SWCNT photocatalyst having a Pt(II) complex on the shell.

**Figure 2:**
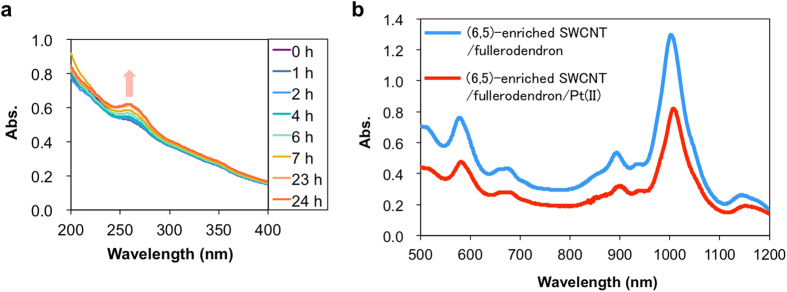
Introduction of Pt(II) complexes into the shell moieties of the coaxial photocatalysts. (**a**) Time-dependent UV-vis spectroscopic data obtained after mixing K_2_PtCl_4_ with (6, 5)-enriched SWCNT/fullerodendron. The absorbance at 255 nm gradually increased because of ligand-to-metal charge transfer between fullerodendron and Pt(II). (**b**) vis-NIR spectra of (6, 5)-enriched SWCNT/fullerodendron and (6, 5)-enriched SWCNT/fullerodendron/Pt(II).

In order to obtain a photosensitizer/co-catalyst interconnecting system that exhibits H_2_ evolution activity under irradiation of light of wavelengths over 600 nm, SWCNT/fullerodendron/Pt(II) coaxial nanohybrids having (6, 5), (7, 5), and (8, 3)SWCNTs cores were fabricated by complexation between K_2_PtCl_4_ and SWCNT/fullerodendron supramolecular nanocomposites based on (6, 5)-enriched SWCNTs according to the literature procedure (Fig. 1)^17^. Complex formation between SWCNT/fullerodendron and platinum(II) can be monitored by the time course of UV-vis spectral changes during the reaction (Fig. 2a). Absorbance at 255 nm increased gradually up to 24 h because of ligand-to-metal charge transfer (LMCT) from the tertiary amine of the dendron moiety to Pt^2+ ^17,26. From a comparison of the absorption bands (500–1200 nm) arising from the SWCNTs before and after the complexation, absorptions decreased slightly because the dispersion of the hybrids was diluted via the complexation process (Fig. 2b). However, the population of the chiralities, e.g., the ratio of absorptions at 570, 650, and 680 nm from the (6, 5), (7, 5), and (8, 3)SWCNTs, respectively, was not changed by the complexation.

**Figure 3:**
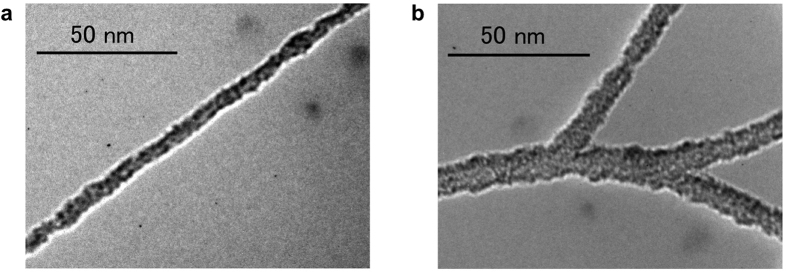
Thin nanofiber structures of coaxial photocatalysts. (**a**,**b**) TEM images of (6, 5)-enriched SWCNT/fullerodendron (**a**) and of (6, 5)-enriched SWCNT/fullerodendron/Pt(II) (**b**).

Transmission electron microscopy (TEM) was used to characterize the photosensitizer/co-catalyst interconnecting system based on SWCNT/fullerodendron supramolecular nanocomposites (Fig. 3). The high-resolution TEM image in Fig. 3a reveals that the supramolecular nanocomposite exhibits nanofibrous morphology similar to that of HiPco/fullerodendron nanocomposites^12^. Interestingly, tiny nanoparticles with a size of around 1 nm, which could be C_60_, were observed on the surface of the nanowires with a thickness of 5 nm. After the complexation of Pt(II), we observed thicker branched fibrous morphology with a diameter of 7–13 nm (Fig. 3b). From these observations, we surmised that limited agglomeration of the SWCNT/fullerodendron supramolecular nanocomposites occurred after the treatment with K_2_PtCl_4_. However, the Pt(II) complex of SWCNT/fullerodendron is still dispersible in water. This finding is consistent with the results of atomic force microscopy (AFM) measurements (Supplementary Fig. 1). The height profiles reveal that the thickness of (6, 5)-enriched SWCNT/fullerodendron/Pt(II) (*ca.* 4.9 nm) is higher than that of (6, 5)-enriched SWCNT/fullerodendron (*ca.* 3.1 nm).

In order to clarify the oxidation state of the shell-anchored Pt complexes, X-ray photoelectron spectroscopy (XPS) measurements of the (6, 5)-enriched SWCNT/fullerodendron/Pt(II) coaxial nanohybrids were conducted (Supplementary Fig. 2). The Pt(4f_5/2_) and Pt(4f_7/2_) peaks are present at 77.3 and 74.0 eV, respectively, which are consistent with the peaks of the Pt(II) complex of the poly(amidoamine) dendrimer reported by Crooks^27^. Hence, we confirmed that coordination of the dendron units of SWCNT/fullerodendron with Pt(II) cause the formation of SWCNT/fullerodendron/Pt(II).

**Figure 4:**
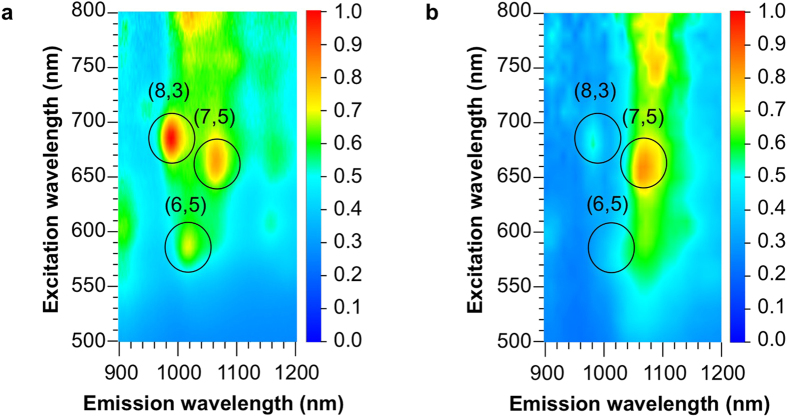
Individuality of SWCNT core of coaxial photocatalysts. (**a**,**b**) Three-dimensional fluorescence spectra of (6, 5)-enriched SWCNT/fullerodendron (**a**) and (6, 5)-enriched SWCNT/fullerodendron/Pt(II) (**b**). Before formation of the fullerodendron-Pt(II) complexes, the PL intensities of (8, 3) and (7, 5) SWCNTs were very high owing to existence of the individual SWCNT. After formation of the fullerodendron-Pt(II) complexes, quenching of PL emission from (6, 5) and (8, 3) SWCNTs was observed because of electron transfer from (6, 5) and (8, 3) SWCNTs to C_60_.

**Figure 5:**
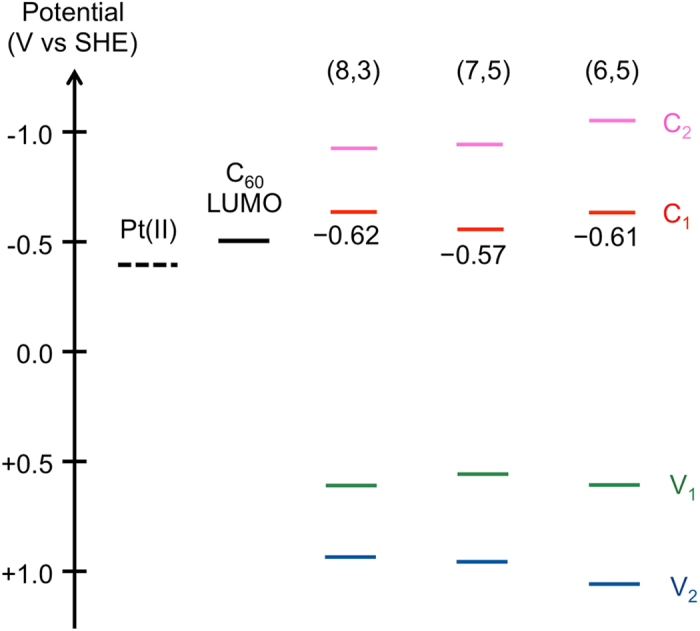
Energy level diagram of (6, 5)-enriched SWCNT/fullerodendron/Pt(II) coaxial photocatalyst system. Energy level diagram of conduction bands (C1 and C2) and valence bands (V1 and V2) of (8, 3), (7, 5), and (6, 5) SWCNTs, LUMO of C_60_, and expected energy level of Pt(II) complexes.

Figure 4 shows three-dimensional photoluminescence (PL) intensity mapping of SWCNT/fullerodendron and SWCNT/fullerodendron/Pt(II) in D_2_O solutions. Before complexation of SWCNT/fullerodendron with K_2_PtCl_4_, three intense peaks can reasonably be assigned to (6, 5), (7, 5), and (8, 3) SWCNTs (Fig. 4a). Although (6, 5) SWCNT exhibited the strongest absorption among these chiralities (Fig. 2b), its PL intensity was weakest because strong bundles exclusively consisting of (6, 5) SWCNT were formed via rebundling process^28^ of its enrichment procedure and were incorporated into the core of the SWCNT/fullerodendron supramolecular nanocomposite. In contrast, the PL intensities of (8, 3) and (7, 5) SWCNTs were very high owing to existence of the individual SWCNT at the core of the coaxial nanowires. Interestingly, after the formation of SWCNT/fullerodendron/Pt(II), quenching of PL emission from (6, 5) and (8, 3) SWCNTs was observed in contrast to the strong luminescence from (7, 5) SWCNT (Fig. 4b). An energy level diagram of the conduction bands (C1 and C2) and valence bands (V1 and V2) of different (*n, m*)SWCNTs along with the LUMO of C_60_ and expected energy level of Pt(II) is shown in Fig. 5. Energy levels of SWCNTs^19^ were corrected by the use of optical band-gap energies estimated by PL spectra, because PL peak positions are substantially red-shifted after the attachment of fullerodendrons on the lateral surface of SWCNTs^18^. The LUMO level of C_60_ was assumed using the reduction potential of fullerodendrimer (E^1^_red_ = −1.12 V vs Fc/Fc^+^)^29^. The LUMO offset between SWCNTs and C_60_ affects the efficiency of the electron transfer from SWCNTs to C_60_^22^. Because the C1 energy level of (7, 5) SWCNT is lower than that of (6, 5) and (8, 3) SWCNTs, PL emission quenching from (7, 5) SWCNT was not observed, which is in marked contrast to that from (6, 5) and (8,3)SWCNTs.

**Figure 6:**
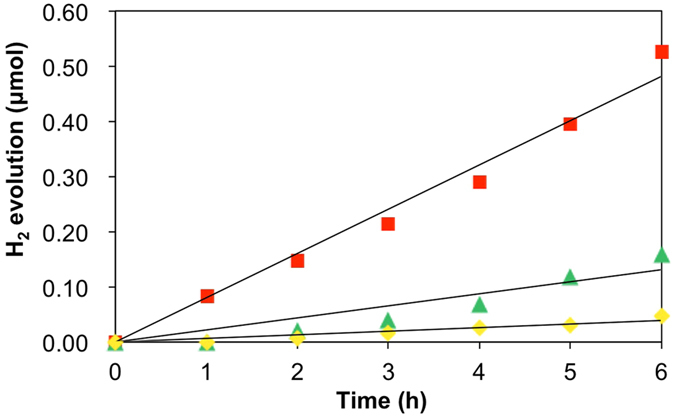
Photocatalytic hydrogen evolution using (6, 5)-enriched SWCNT/fullerodendron/Pt(II) coaxial photocatalysts under monochromatic light. Time dependencies of H_2_ evolution from water by use of (8, 3)SWCNT photocatalyst (◾), (7, 5) SWCNT photocatalyst (◆), and (6, 5) SWCNT photocatalyst (▲). The overall quantum yields for H_2_ evolution were 1.5% (for (8, 3) SWCNT photocatalyst), 0.17% (for (7, 5) SWCNT photocatalyst), and 0.35% (for (6, 5) SWCNT photocatalyst).

In order to clarify the effect of chirality of SWCNTs on the efficiency of photocatalytic H_2_ evolution, we investigated the photocatalytic activity of SWCNT/fullerodendron/Pt(II) upon chirality-selective photo-excitation by the use of monochromatic light irradiation at 570, 650, and 680 nm, which are the E_22_ absorptions of (6, 5), (7, 5), and (8, 3) SWCNTs, respectively. In a typical experiment, 150 mL of aqueous solution of SWCNT/fullerodendron/Pt(II) hybrids (SWCNT content 0.025 mg), Tris-HCl buffer (pH 7.5, 0.12 mM), and benzyldihydronicotinamide (BNAH; 1.2 mM), was exposed to monochromatic light (570, 650, or 680 nm) using a 300 W Xenon arc lamp with bandpass filters while being stirred vigorously at 25 °C. After the designated period, the gas phase above the solution was analyzed by gas chromatography. Figure 6 (◾) shows plots of the total amount of H_2_ produced *versus* time using monochromatic light irradiation at 680 nm. A steady generation of H_2_ (0.083 μmol/h) was observed without an induction period or a decrease in activity during 6 h of irradiation. Compared with the H_2_ generated by the use of monochromatic light irradiation at 570 or 650 nm, 0.022 μmol/h (Fig. 6 (▲) or 0.0065 μmol/h (Fig. 6 (◆)), respectively, the amount of H_2_ evolution under 680 nm irradiation was highest (0.083 μmol/h, Fig. 6 (◾)). Furthermore, in order to compare the efficiency of photocatalytic H_2_ evolution between (6, 5), (7, 5), and (8, 3) SWCNTs, we evaluated quantum yields by the use of monochromatic light irradiation at 570, 650, and 680 nm. The overall quantum yields for H_2_ evolution (QY = 2 × number of H_2_ molecules generated / number of photons absorbed) were 0.35% (for (6, 5) SWCNT/fullerodendron/Pt(II)), 0.17% (for (7, 5) SWCNT/fullerodendron/Pt(II)), and 1.5% (for (8,3)SWCNT/fullerodendron/Pt(II)). These quantum yields are consistent with the PL intensities and emission quenching shown in Fig. 5. Although the charge-recombination occurred between C_60_ and SWCNT in (7, 5) SWCNT/fullerodendron/Pt(II) causes not only strong emission but also low efficiency of H_2_ generation, efficient electron transfer from C_60_ to Pt(II) in (8, 3) SWCNT/fullerodendron/Pt(II) gives rise to the fluorescence quenching and H_2_ evolution.

This result indicated that both the chirality and the individuality of the SWCNT core of the coaxial photosensitizer affect the efficiency of photocatalytic H_2_ evolution. It is noteworthy that SWCNT/fullerodendron/Pt(II) showed a quite high quantum yield of 1.5% under 680 nm light irradiation, which is to the best of our knowledge, the highest quantum yield for H_2_ evolution using a nanocarbon/co-catalyst interconnecting system under an illumination wavelength of over 600 nm.

In summary, we demonstrated photocatalytic hydrogen evolution from water using SWCNT/fullerodendron nanohybrids with the help of a sacrifice donor, BNAH. Upon chirality-selective photo-excitation by monochromatic light irradiation at 680 nm (E_22_ absorption of (8, 3) SWCNT), we provided the first clear-cut example of a H_2_ evolution reaction photosensitized by SWCNT. Furthermore, efficiency of the photocatalytic reaction was affected not only by the individuality but also by the chiral indices (*n, m*) of the SWCNT core of the nanocoaxial photocatalysts. These findings offer the possibility of an efficient hydrogen evolving system under illumination at wavelengths longer than 600 nm by employing a combination of SWCNTs with appropriate chiralities. From the viewpoint of utilization of exciton dissociation in SWCNT heterojunctions, synergistic development between a SWCNT/C_60_ photocatalyst system in solution and a SWCNT/C_60_ photovoltaic system in thin film is highly anticipated. Further studies of SWCNT photocatalysts are in progress, not only to explore the kinetics and energetics of photoinduced electron transfer but also to make photocatalytic systems more efficient and useful.

## Methods

### Materials and methods

Absorption data were recorded on a Shimadzu UV-3150 spectrophotometer using a standard cell with a path length of 10 mm. TEM measurements for (6, 5)-enriched SWCNT/fullerodendron/Pt(II) nanocomposites were conducted using a JEM-2100 transmission electron microscope (80 kV). The specimens for the measurements were prepared by applying a few drops of sample solution onto a holey carbon-coated copper grid, and then evaporating the solvent. Atomic force microscopy (AFM) observation was carried out using a Seiko SPA 400-DFM. Samples for observation were prepared by placing a drop of the aqueous specimen on freshly cleaved mica, then allowing each drop to dry. X-ray photoelectron spectroscopy (XPS) measurements were conducted with a JPS-9030 spectrometer using a monochromatic Al Kα X-ray source with a pass energy of 20 eV. The spectra were adjusted with reference to the C 1 s peak at 284.6 eV. Three-dimensional fluorescence spectra data were obtained using a spectrofluorometer (Shimadzu, NIR-PL system). (6, 5)-enriched SWCNTs were purchased from Sigma-Aldrich Co. All other reagents were purchased from Kanto Kagaku Co., Ltd, Sigma-Aldrich Co., and Tokyo Kasei Co., Ltd. All chemicals were used as received. Fullerodendron was prepared according to the reported procedure^29^.

### Preparation of photocatalyst solution

(6, 5)-enriched SWCNTs (1.0 mg) were placed in a water solution (10 mL) of fullerodendron (25.5 mg, 0.01 mmol) and then sonicated with a bath-type ultrasonic cleaner (Honda Electronics Co., Ltd., vs-D100, 110 W, 24 kHz) at 17–25 °C for 4 h. After the suspension was centrifuged at 3000 G for 30 min, a black supernatant dispersion, which included excess fullerodendrons and (6, 5)-enriched SWCNT/fullerodendron supramolecular nanocomposites, was collected. The (6, 5)-enriched SWCNT/fullerodendron nanocomposite was purified by dialysis for 3 days using dialysis tubing (SPECTRUM RC MEMBRANES Pro 4) to remove excess fullerodendrons. The dialysis process was continued until the dialysate showed no change in absorption at 255 nm in UV-vis spectra. Then, 1.0 mL of the stock solution of (6, 5)-enriched SWCNT/fullerodendron nanocomposites was diluted with 19.0 mL of deionized water and used for the following experiment. The aqueous solution of (6, 5)-enriched SWCNT/fullerodendron nanocomposites was added to potassium tetrachloroplatinate(II) (0.28 mg, 0.68 μmol) in deionized water (0.28 mL) and stirred at 50 °C for 24 h to obtain a solution of (6,5)-enriched SWCNT/fullerodendron/Pt(II) coaxial nanowires.

### Hydrogen evolution

An aqueous solution of Tris-HCl buffer (3.5 mL, pH 7.5, 5 mM), (6, 5)-enriched SWCNT/fullerodendron/Pt(II) nanohybrids (5.0 mL), BNAH (38.6 mg, 1.20 mM), and deionized water (145 mL) in a Pyrex reactor was degassed for five cycles and purged with Ar. Upon vigorous stirring, the solution was irradiated with a 300 W Xenon arc light (Ushio model UXL-500 W) through bandpass filters (570, 650, and 680 nm: ASAHI SPECTRA CO, M. C.). After a designated period of time, the cell containing the reaction mixture was connected to a gas chromatograph (Shimadzu, TCD, molecular sieve 5 A: 2.0 m × 3.0 mm, Ar carrier gas) to measure the amount of H_2_ above the solution. The apparent quantum yield (Φ_H2_) is defined as follows. Φ_H2_ = number of H_2_ molecules generated × 2/number of photons absorbed, which was evaluated from a change in the power of the transmitted light, measured using a power meter (Photo-Radiometer Model HD 2302.0 coupled with an irradiance measurement probe LP 471 RAD having an exposure window diameter of 1.6 cm) placed behind the cell parallel to the irradiation cell face.

## Additional Information

**How to cite this article:** Murakami, N. *et al*. SWCNT Photocatalyst for Hydrogen Production from Water upon Photoexcitation of (8, 3)SWCNT at 680-nm Light. *Sci. Rep.* **7**, 43445; doi: 10.1038/srep43445 (2017).

**Publisher's note:** Springer Nature remains neutral with regard to jurisdictional claims in published maps and institutional affiliations.

## Supplementary Material

Supplementary Information
