# Supplementary material for: SWCNT Photocatalyst for Hydrogen Production from Water upon Photoexcitation of (8, 3) SWCNT at 680-nm Light
Source: Sci Rep. 2017 Mar 6;7:43445. doi: 10.1038/srep43445 (PMC5337977; doi:10.1038/srep43445)
Supplement: Supplementary Information [file srep43445-s1.pdf]

Supplementary information

SWCNT Photocatalyst for Hydrogen Production from Water upon Photoexcitation of (8,3)SWCNT at 680-nm Light

Noritake Murakami,[1] Yuto Tango,[1] Hideaki Miyake,[2] Tomoyuki Tajima,[1] Yuta Nishina,[3] Wataru Kurashige,[4] Yuichi Negishi,[4] and Yutaka Takaguchi[1]*

[1]*Graduate School of Environmental and Life Science; Okayama University;*

3-1-1 Tsushima-Naka; Kita-ku, Okayama 700-8530; Japan

[2]*Graduate School of Sciences and Technology for Innovation; Yamaguchi University;*

2-16-1 Tokiwadai; Ube, Yamaguchi 755-8611; Japan

[3]*Research Core for Interdisciplinary Sciences; Okayama University; 3-1-1 Tsushima-Naka;*

Kita-ku, Okayama 700-8530; Japan

[4]*Department of Applied Chemistry; Faculty of Science Division I;*

Tokyo University of Science; 1-3 Kagurazaka; Shinjuku-ku, Tokyo 162-8601; Japan

Contents: Page:

Supplementary Figure 1 2

Supplementary Figure 2 3

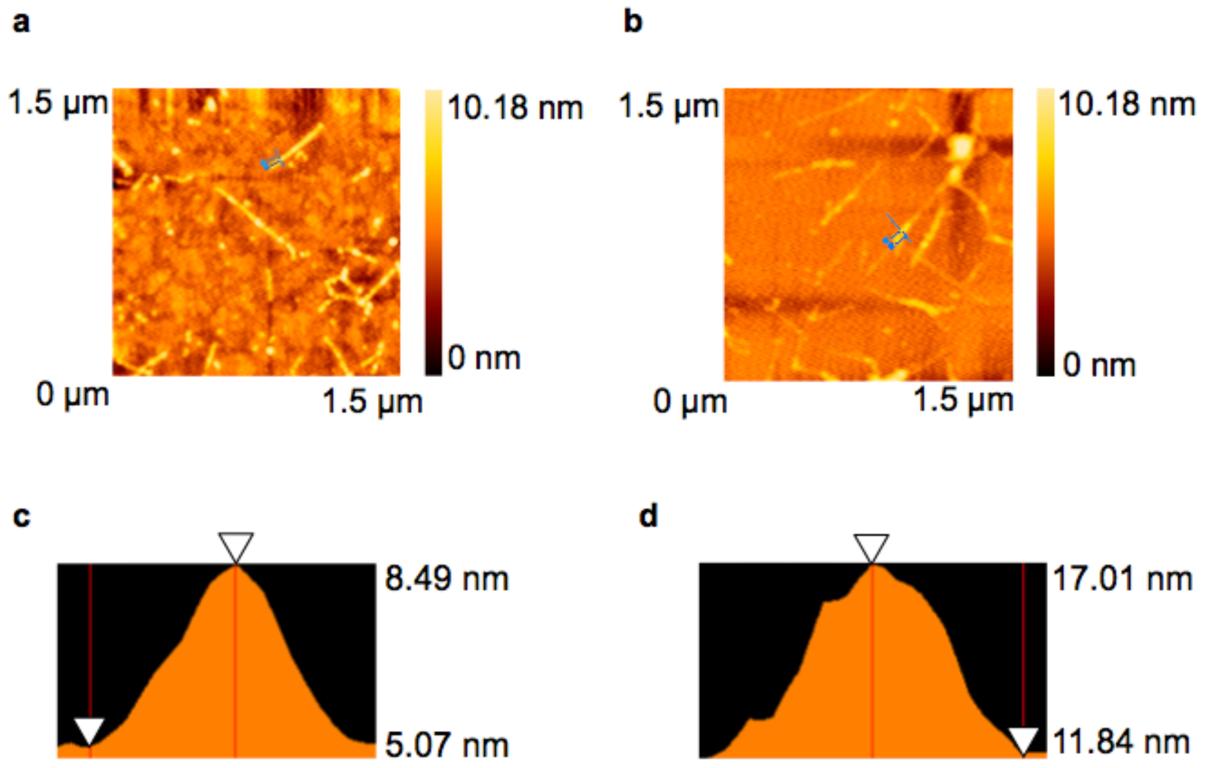

Supplementary Figure 1 | Thin nanofiber structures of coaxial photocatalysts. a, **b**, AFM images of (6,5)-enriched SWCNT/fullerodendron (**a**) and (6,5)-enriched SWCNT/fullerodendron/Pt(II) (**b**). **c**, **d**, AFM topographic line profiles in **a** and **b**, respectively.

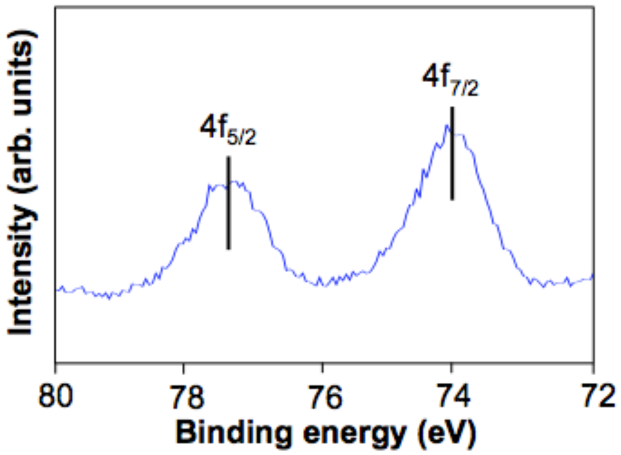

Supplementary Figure 2 | Clarifying the oxidation state of shell-anchored Pt complexes. XPS spectra of (6,5)-enriched SWCNT/fullerodendron/Pt(II). The Pt($4f_{5/2}$) and Pt($4f_{7/2}$) peaks present at 77.3 and 74.0 eV, respectively, are consistent with the peaks of the Pt(II) complex.
